# Knowledge and practice of infant exposure to sunlight among mothers in the rural villages of Mettu district, southwest Ethiopia

**DOI:** 10.3389/fpubh.2023.1166976

**Published:** 2023-07-13

**Authors:** Askalemariam Tadesse, Solomon Yeshanew, Abdi Geda Gedefa

**Affiliations:** ^1^Mettu Karl Referral Hospital, Mettu, Ethiopia; ^2^Department of Biology, Debre Markos University, Debre Markos, Ethiopia; ^3^Department of Public Health, Mettu University, Mettu, Ethiopia

**Keywords:** infants, knowledge, Mettu district, practice, sunlight exposure

## Abstract

**Background:**

Sunlight is essential for the synthesis of vitamin D and supports bone growth and strength. The awareness of mothers about the benefit of exposing their infants to sunlight, as well as the practice of doing so, is important to a child’s proper growth and development. The current study looked into mothers’ knowledge, practice, and factors related to infant sunlight exposure in rural villages in the Mettu district, southwest Ethiopia.

**Methods:**

A community-based cross-sectional study design was utilized with randomly selected mothers of newborns from rural areas in the Mettu district. A structured questionnaire was used to collect information. Analysis of the data was performed using the SPSS version 21 software. Both descriptive and inferential analyses were carried out, and *p* < 0.05 was considered statistically significant.

**Results:**

Among the 600 mothers who participated in the present study, 397 (66.2%) had good knowledge and 346 (57.7%) practiced proper exposure of their infants to sunlight. Out of the 482 mothers who practiced sunning, 382 (79.3%) did it daily, and 409 (84.8%) began sunning before 45 days of birth. However, 453 (94%) of the mothers used lubricants while sunbathing. Sociodemographic characteristics were found to have statistically significant associations with the level of knowledge and practice of mothers. Mothers with four to six children [AOR = 0.511, 95% CI: (0.352–0.741)] and those who got the information from health professionals [AOR = 3.285, 95% CI: (2.154, 5.011)] for the knowledge level, and mothers aged 36 years old and above [AOR = 0.801, 95% CI: (0.533, 0.161)], who were married during the data collection period [AOR = 0.370, 95% CI: (0.155, 0.884)], and employed by the government [AOR = 4.081, CI: (1.477, 11.280)] for the practice level were found to be significantly (*p* < 0.05) associated with the level of infant sunning.

**Conclusion:**

Despite the fact that the majority of mothers in the study area had good knowledge and practice of infant sunlight exposure, there are clear signs that further work is needed to narrow the large gap seen in the present findings. Thus, district and zonal health offices, as well as stakeholders working with children, should start periodical maternity health education, and professional development training for health post workers.

## Background

The full solar spectrum is essential for good health and well-being. Humans are physiologically adapted to produce vitamin D in response to Ultra Violet B Radiation (UVB) from the sun. Other parts of the spectrum appear to be beneficial as well. Vitamin D is a fat-soluble vitamin that regulates calcium absorption in the body, thereby contributing to bone health (1). Its synthesis in the skin is dependent on exposure to sunlight (2). Diet and supplements are also other sources of vitamin D (3, 4).

Vitamin D has numerous health benefits, including bone strengthening, which helps to prevent rickets in children and osteomalacia in adults. It can also slow the growth of some cancerous cells (5, 6). Visible sunlight to the eyes provides health benefits by influencing the timing of melatonin synthesis, preserving normal and robust cardiac rhythms, and lowering the risk of seasonal affective disorder (7). Furthermore, it prevents Low Birth Weight (LBW), birth asphyxia, and deafness caused by premature birth (8). A daily exposure of 10 min naked and 30 min dressed is enough to cause vitamin D synthesis in the body (9, 10). However, it is also critical to limit skin exposure to excessive Ultra Violet Radiation (UVR) (11–13).

The most important cause of vitamin D deficiency is a lack of understanding that moderate sun exposure is the primary source of vitamin D for most members of the community across the globe, particularly in Africa (14). In Africa, more specifically in the sub-Saharan region, rickets is a common public health problem although it is usually caused by mixed vitamin D and calcium deficiencies (15). Similarly, in Ethiopia, rickets caused by vitamin D deficiency have also been reported (16). Indeed, in the early 1960s, health education to change maternal behavior and sunning infants were adopted as the principal strategy to combat rickets in the country. However, the implementation of the policy has been inconsistent, and health messages have lacked focus on factors that influence maternal practice, preventing infants from getting enough sunlight (17). This was significant due to a lack of adequate information on the determinants of this specific risk behavior among Ethiopian mothers (18–24).

A recent systematic review and meta-analysis on the subject of determining the level of awareness and practice of mothers toward sunning children in Ethiopia found it to be quite low. Mothers’ overall estimated good level of knowledge and practice regarding infant sunlight exposure was found to be 56.08 and 55.63%, respectively (24). This means that more research and interventions are needed, particularly among people in rural areas, to address the country’s growing public health concerns about vitamin D-deficiency rickets. Hence, the current study assessed mothers’ knowledge and practice of infant sunlight exposure in rural villages of Mettu district, southwest Ethiopia.

## Materials and methods

### Study design and setting

The community-based cross-sectional study design was employed in the rural villages of the Mettu district, southwest Ethiopia from June to August 2019. The study area is located in the Ilubabor administrative zone, which is approximately 600 km southwest of the capital Addis Ababa. It covers an area of 1,452 km^2^ and an altitude ranging from 1,000 to 2,027 m above sea level. During the data collection period, the minimum and maximum temperatures of the district were 18.5°C and 31°C, respectively. According to the 2007 national census report, the district comprises 30 Kebeles with an estimated resident population of 61,954 (30,982 men and 30,972 women).^1^ During the data collection period, the number of infants was estimated to be 2,732 in the district (Mettu district annual health report).

### Study population

Mothers who had infants aged less than 1 year during the data collection period were our target population. However, mothers who had infants and came from outside of the study area as a guest during the study period, mothers who started residence less than 6 months, and who were unable to speak and were severely ill were excluded from the study.

### Sample size determination and sampling procedure

**Figure 1:**
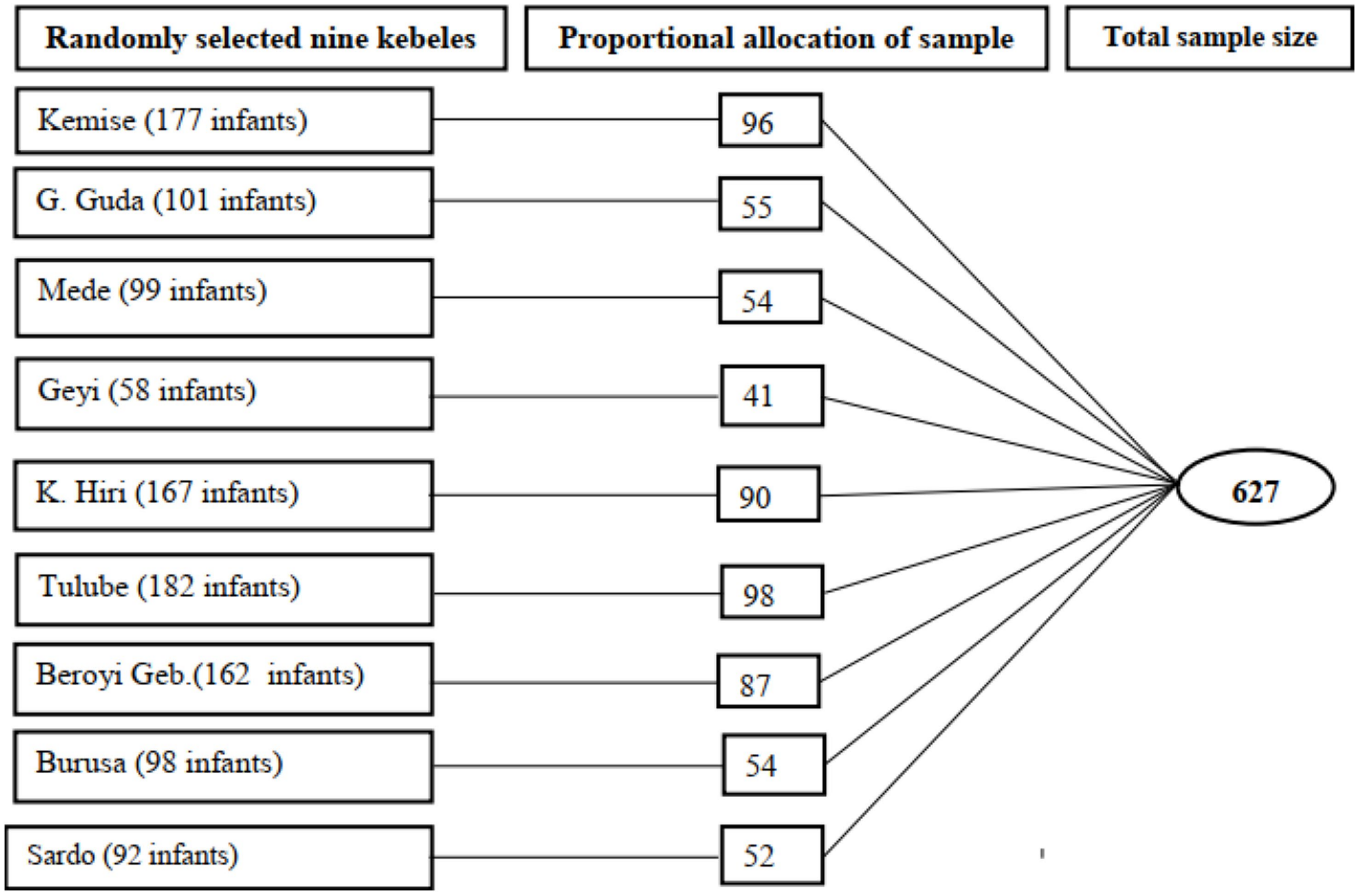
Schematic presentation of a sampling frame of study participants, Mettu district, southwest Ethiopia, 2019.

The sample size was determined using the single population proportion formula [*n* = *Z*α/2^2^ × *p* × (1-*p*)/*d*^2^], considering the nationally reported levels of knowledge and practice of infant sunning among mothers in Ethiopia was nearly 55.8% (24). At a 95% confidence interval, 0.05 margins of error, and 10% non-response rate, the total sample size was 418. However, a design effect of 1.5 was considered to correct the loss of sampling efficiency resulting from the use of multi-stage sampling, and the final estimated sample size was 627. Nine kebeles, nearly 30% of the 30 rural kebeles of the district were selected randomly. Then, study participants were recruited using proportional allocation by taking approximately 55% of the total number of infants in each village except for the “Geyi” village, where we took nearly 70% of the total population due to the small number of infants reported in the village during the data collection period (Figure 1). Finally, in each village, a list of infants was prepared by the health post of the village, and randomly selected mothers were subjected to an interview.

### Operational definitions

#### Adequate sunlight exposure

Mothers who exposed their infants to sunshine in the morning time (8:00–10:00 a.m.) for 10 min unclothed or 30 min clothed daily for the last 3 months starting before 45 days of birth.

#### Knowledge

Ten questions were used to assess mothers’ awareness of infant sunlight exposure. The median score for these knowledge questions was six, and mothers with scores higher than the median were regarded to have good knowledge, while those with scores lower than the median were considered to have poor knowledge about infant sunlight exposure.

#### Practice

Twelve questions were used to assess mothers’ level of practice about infant sunlight exposure. The median score for these practice questions was seven, and mothers with scores above the median were considered to have a good practice, while those with scores below the median were regarded to have poor practice with infant sunshine exposure.

### Data collection tools and procedures

After reviewing several works of the literature, a structured questionnaire was prepared following the Ethiopian Demographic and Health Survey (EDHS) format with minor modifications. Face-to-face interviews were employed to gather data, with an emphasis on socioeconomic and demographic characteristics, sources of information about infant sunlight exposure, mothers’ fear, and other variables of mothers’ knowledge and practice regarding infant sunlight exposure. Similarly, the questionnaire had several queries that are concerned with factors associated with the knowledge and practice of mothers’ infant sunbathing. Knowledge questions were yes and no types while the practice questions were yes/no/sometimes types. The knowledge score ranged from 0 to 10 points (Yes = 1, and No = 0). Thus, an individual who scored less than or equal to 50% (≤5 scores) was considered poor, and those who scored more than 50% (>5 scores) were considered good knowledge. Similarly, the practice score ranged from 0 to 12 points (Yes = 2, Sometimes = 1, and No = 0), and a score >6 was considered good and a score ≤6 was considered poor practice. Data were collected by trained health extension workers (HEWs) who were supervised by senior nurse professionals.

### Data quality management

The questionnaire was translated and back-translated (English/Affan Oromo, and English/Amharic) by two bilingual experts. Then, two senior faculty members validated the questionnaire. Data collectors and supervisors received a one-day data collection training. In order to assess the convenience and interpretation of the questionnaire, a pilot test was conducted in a nearby district with 32 (5%) participants, and the reliability of the questionnaire was 0.78 (Cronbach’s alpha). Any necessary adjustments were made before the actual data collection, and finally, the collected data were checked for completeness and internal consistency.

### Data analysis

Data was cleaned and entered into Epi Data version 3.1, and exported to SPSS version 20 software for descriptive and inferential analysis. Binary logistic regression was considered to assess the effect of independent variables on the dependent variables. The *p*-value of a bivariate analysis of less than 0.25 was exported for multivariate regression analysis to determine the predictor variables for the level of knowledge and practice of infant sunning among mothers. Finally, variables with *p* < 0.05 in the final regression were considered statistically significant, and the model fitness was checked by Hosmer-Lemeshow at *p* > 0.05.

## Results

### Sociodemographic characteristics

Six hundred (95.7%) of the 627 recruited participants provided complete information on the study. However, data collected from 27 (4.3%) of the participants were excluded from the analysis due to its incompleteness and inconsistency. Study participants were found with a mean age of 27.38 (SD ± 5.23), and 339 (56.5%) were between 26 and 35 years. The majority (93%) were married, and 93 (15.5%) were unable to read and write. Furthermore, 444 (74%) of the mothers who participated in this study were housewives (Table 1).

**Table 1:** Sociodemographic characteristics of study participants, Mettu district, southwest Ethiopia, 2019 (*n* = 600).

Characteristic		Frequency	Percentage (%)
Age	16–25 years	186	31.0
	26–35 years	339	56.5
	36 and above years	75	12.5
Marital status	Married	558	93.0
	Single	18	3.0
	Others^*^	24	4.0
Educational status	Unable to read/write	93	15.5
	Grade 1–8	373	62.2
	Grade 9–12	114	19.0
	Diploma and above	20	3.3
Occupation status	Housewife	444	74.0
	Merchants	105	17.5
	Government employee	24	4.0
	Farmer	24	4.0
	Others^**^	3	0.5
Family size	<3	358	59.7
	4–6	208	34.6
	>6	34	5.7
Husband’s educational status	Unable to read/write	47	8%
	Grade 1–8	366	62.7%
	Grade 9–12	147	25.2%
	Diploma and above	24	4.1%

*Widowed, Divorced, or Separated; **Private employee, Students, or Drivers.

### Level of knowledge of mothers about sunlight exposure

The majority of the mothers (88.8%) were informed about infant sunlight exposure. Among these mothers who had information about infant sunbathing, 523 (98.1%) of them were aware of its benefit, and 439 (90.3%) of them knew the perfect sunning time to be in the morning. However, only 47 (8.8%) of the mothers who participated in the study had a misconception, with the majority (40.4%) believing that sunning could weaken infants (Table 2).

**Table 2:** Knowledge of mothers about sunlight exposure of their infants in Mettu district, southwest Ethiopia, 2019 (*n* = 600).

Variable	Category	Frequency	Percentage (%)
Do you have information about sunlight exposure	Yes	533	88.8
	No	67	11.2
Knowledge of mothers	Good knowledge	397	66.2
	Poor knowledge	203	33.8
Information of mothers about the benefit of sunlight exposure (*N* = 533)	Yes	523	98.1
	No	10	1.9
Information on the benefit of sunlight exposure (*N* = 523)	Strengthen the bone and teeth	251	47.0
	Increase baby fatness	82	15.7
	Produce vitamin D	25	4.8
	Strengthen the body	104	19.9
	Make the baby sleep	61	11.7
Maternal negative information about sunlight exposure	Yes	47	8.8
	No	486	91.2
Mothers’ misconceptions about sunlight exposure (*N* = 47)	Skin cancer	14	29.8
	Blindness	14	29.8
	Make the baby weak	19	40.4
Mothers’ information on perfect exposure time (*N* = 486)	Morning (8:00–10:00 a.m.)	439	90.3
	Mid-day (11:00–1:00 p.m.)	2	0.4
	Afternoon (2:00–4:00 p.m.)	45	9.3
Information about the mechanism of compensation for sunlight exposure (*N* = 486)	Vitamin D supplement	22	4.5
	Breastfeeding	32	6.6
	Nutritious food	23	4.7
	No compensation	346	71.2
	Warm clothing	18	3.7
	Warm the baby	42	8.6
	Vaccination	3	0.6

### The level of the practice of mothers regarding sunlight exposure

More than half of mothers (57.7%) who participated in the study practiced infant sunning. Among the 533 (88.8%) mothers who were informed about infant sunlight exposure, 482 (90.4%) of them exposed their infants to sunlight properly, and 409 (84.8%) began before 45 days of birth, with the majority (79.3%) doing so daily. However, 94% of mothers who exposed their infants to sunlight appear to have applied various types of lubricants (Table 3).

**Table 3:** Practice of mothers on sunlight exposure of their infants in Mettu district, southwest Ethiopia, 2019 (*n* = 600).

Variables	Category	Frequency (*n*)	Percentage (%)
Level of practice	Good practice	346	57.7
	Poor practice	254	42.3
The habit of infant exposure to sunlight (*N* = 533)	Yes	482	90.4
	No	51	9.6
Sunlight exposure start time (*N* = 482)	Before 15 days	45	9.3
	16–30 days	176	36.5
	31–45 days	188	39.0
	After 45 days	73	15.2
Frequency of sunlight exposure (*N* = 482)	Daily	382	79.3
	Sometimes	100	20.7
Place of exposure (*N* = 482)	Outdoor	461	95.6
	Indoor	21	4.4
Total sunlight exposure duration (*N* = 482)	4 months	103	21.4
	6 months	287	59.5
	9 months	86	17.8
	1 year	6	1.3
Condition of clothing during exposure (*N* = 482)	Unclothed	368	76.3
	With diapers	8	1.7
	Partly covered	82	17.0
	Complete covered	24	5.0
Length of exposure (*N* = 482)	5–10 min	117	24.3
	10–15 min	242	50.2
	15–30 min	101	20.9
	>30 min	22	4.6
Lubricants usage during exposure (*N* = 482)	Yes	453	94.0
	No	29	6.0
Time of lubricant application (*N* = 453)	Before exposure	25	5.5
	During exposure	377	83.2
	After exposure	51	11.3
Types of lubricant used (*N* = 453)	Baby Vaseline	20	4.4
	Baby lotion	23	5.1
	Butter	399	88.1
	Others	11	2.4

### Factors associated with the level of knowledge of mothers about infant sunning

To identify major responsible factors associated with mothers’ knowledge and practice level regarding infant sunlight exposure, a binary logistic regression analysis was performed. Marital status, family size, educational status, occupation, sources of information, and husband’s educational status were found to be predictors of knowledge level. Because bivariate logistic regression ignores the relationship between independent variables, we used multivariate analysis to reach a valid conclusion about the population parameter. Candidates for multivariate analysis were variables with *p* < 0.25 level of significance in bivariate analysis. Thus, the size of the family and source of information was found to be significantly (*p* < 0.05) associated to mothers’ knowledge of sunning their infants. As a result, mothers with less than three children were used as a reference, and their odds of knowing the importance of infant sunlight exposure were 49% lower than those of mothers with four to six children [AOR = 0.511, 95% CI: (0.352–0.741)]. Mothers with six or more children were 40% more likely to be knowledgeable than mothers with three or fewer [AOR = 0.400, 95% CI: (0.192, 0.833)]. Moreover, mothers who received information about the importance of infant sunlight exposure from health professionals and HEWs were 3.3 times [AOR = 3.285, 95% CI: (2.154, 5.011)] and 2.1 times [AOR = 2.147, 95% CI: (1.173, 3.929)] more likely to be knowledgeable than those who received information from neighbors, respectively (Table 4).

**Table 4:** Factors associated with knowledge of mothers about sunlight exposure of infants in Mettu district, southwest Ethiopia, 2019 (*n* = 600).

Variables	Category	Knowledge level				
		Poor knowledge	Good knowledge	COR [95% CI]	AOR [95% CI]	*p*-value
Marital status	Married	182	376	1		
	Single	9	9	0.484 (0.189, 1.240)	0.472 (0.163, 1.368)	0.167
	Others	12	12	0.484 (0.213, 1.098)	0.435 (0.163, 1.157)	0.095
Maternal education	Unable to read/write	3	58	1		
	Grade 1–8	115	258	1.345 (0.843, 2.174)	1.348 (0.744, 2.442)	0.325
	Grade 9–12	46	68	0.892 (0.508, 1.565)	0.9459 (0.446, 2.002)	0.882
	Diploma and above	7	13	1.121 (0.408, 3.077)	1.630 (0.244, 10.886)	0.614
Family size	<3	97	261	1		
	4–6	89	119	0.497 (0.347, 0.712)	0.511 (0.352, 0.741)	0.001^*^
	>6	17	17	0.372 (0.182, 0.757)	0.400 (0.192, 0.833)	0.014^*^
Husband’s education	Unable to read/write	23	26	1		
	Grade 1–8	120	257	1.895 (1.038, 3.457)	1.632 (0.874, 3.3004)	0.124
	Grade 9–12	51	99	1.717 (0.892, 3.306)	1.298 (0.654, 2.578)	0.456
	Diploma and above	9	15	1.474 (0.543, 4.003)	1.460 (0.429, 4.971)	0.545
Occupation	Housewife	146	298	1		
	Gov’t employee	9	15	0.817 (0.349, 1.910)	0.814 (0.274, 2.419)	0.712
	Farmers	13	11	0.415 (0.181, 0.948)	0.536 (0.230, 1.251)	0.149
	Merchant	34	71	1.023 (0.650, 1.611)	0.981 (0.617, 1.559)	0.934
	Others	1	2	0.980 (0.88, 10.895)	0.705 (0.63, 7.912)	0.777
Information sources	Neighbor	98	78	1		
	HEWs	26	44	2.126 (1.204, 3.755)	2.147 (1.173, 3.929)	0.013^*^
	Health profession	75	199	3.334 (2.238, 4.967)	3.285 (2.154, 5.011)	0.001^*^
	TV/radio	4	9	2.827 (0.839, 9.525)	2.107 (0.622, 7.137)	0.231

*p < 0.05; COR, Crude odd ratio; AD, Adjusted odd ratio; CI, Confidence of interval.

### Factors associated with the level of the practice of mothers on infant sunning

Maternal age, marital status, family size, occupation, and information source were identified as candidate predictor variables for multivariate analysis. Thus, mothers under the age of 24 had a 47% lower chance of having good practice than mothers aged 35 and above [AOR = 0.533, 95% CI] (0.309, 0.921). Married women were 2.7 times more likely to practice infant sunning than divorced and widowed [AOR = 2.699, 95% CI (1.131, 6.442)]. Mothers who worked for the government were also found to be 4.0 times more likely to have good practice than housewives [AOR = 4.081, 95% CI (1.477, 11.280)] (Table 5).

**Table 5:** Factors associated with the practice of mothers toward sunlight exposure of infants in Mettu district, southwest Ethiopia, 2019 (*n* = 600).

		Practice level				
Variables	Category	Poor practice	Good practice	COR [95% CI]	AOR [95% CI]	*p*-value
Mother’s age	18–24 years	69	117	1		
	25–34 years	145	194	0.789 (0.547, 1.139)	0.801 (0.553, 1.161)	0.241
	35 and above years	40	35	0.516 (0.300, 0.888)	0.533 (0.309, 0.921)	0.024^*^
Marital status	Married	231	327	1		
	Single	7	11	3.143 (0.881, 11.215)	2.897 (0.802, 10.464)	0.105
	Others	16	8	2.831 (1.192, 6.726)	2.699 (1.131, 6.442)	0.025^*^
Family size	1–3	148	210	1		
	4–6	87	121	0.980 (0.693, 1.386)	0.880 (0.590, 1.313)	0.532
	>6	19	15	0.556 (0.274, 1.130)	0.983 (0.414, 2.336)	0.969
Occupation	House wise	192	252	1		
	Gov’t employee	6	18	2.286 (0.890, 5.868)	4.081 (1.477, 11.280)	0.007^*^
	Farmers	8	16	1.524 (0.639, 3.634)	1.400 (0.512, 3.824)	0.512
	Merchants	46	59	0.977 (0.636, 1.500)	1.197 (0.746, 1.919)	0.457
	Others	2	1	0.381 (0.034, 4.232)	0.415 (0.036, 4.730)	0.478
Information sources	Neighbors	76	100	1		
	HEWs	32	38	0.902 (0.517, 1.575)	0.894 (0.500, 1.598)	0.706
	Health professional	140	134	0.727 (0.497, 1.065)	0.695 (0.468, 1.031)	0.071
	TV/radio	6	7	0.887 (0.286, 2.746)	0.947 (0.298, 3.007)	0.926

*p < 0.05; COR, Crude odd ratio; AD, Adjusted odd ratio; CI, Confidence of interval.

## Discussion

The present study assessed the level of knowledge and practice of infant sunning among mothers of the Mettu district in southwest Ethiopia. The findings then revealed that 11.2% were unaware of the importance of infant sunlight exposure, and 42.3% practiced it poorly. This finding supports the growing public health concern of inadequate infant sunlight exposure across the country (18–25). Similarly, reports from Farta district, Gonder (53.98%) (25), Yirgalem, Sidama (5.2%) (23), Debre Markos, Gojam (40%) (21), and Debre Birhan, Shewa (35.3%) (18), found that a significant number of study participants were not aware of the benefits of infants’ exposure to sunlight.

In this study, nearly two-thirds (66.2%) of the mothers had a good knowledge of infant sunlight exposure. The finding is consistent with the report from Aleta Wondo, in which 62.8% of enrolled mothers were found to be knowledgeable (22). A report from Debre Markos town (60%) is in agreement with the current finding as well (21). Similarly, 57.7% of mothers properly exposed their infants to sunlight in the present study, which is consistent with the report from Aleta Wondo (58%) (22). However, the report of the present finding is to be higher than Debre Markos (44.6%) (21) and Yirgalem (54.5%) (23), and lower than Debre Birhan (65.7%) (18) reports. The variation could be due to the differences in sociodemographic (employment status) (23), cultural (fear of evil eye and sunburn “Mich”) (19, 21, 22), and weather conditions (cold air and pneumonia) (19, 21, 23) as explained in the aforementioned reports.

However, the lower level of sunning practice seen in the present report compared to Debre Birhan’s findings could be elucidated by the fact that the majority (77.7%) of study participants of the herein study did not complete secondary education; however, 73.4% of the mothers enrolled in Debre Birhan’s study did (18). The justification could be explained as education could increase the level of communication, confidence, and access to more information which could assist them to have a better understanding of health and related issues.

In terms of negative perception, 8.8% of the mothers who took part in the present study believed that sunbathing could harm their children. Among these mothers, 40.4% assumed that sunning could weaken the children, while 29.8% assumed it could cause blindness. The present finding was discovered to be consistent with reports from Debre Birhan (18), Yirgalem (23), and Aleta Wondo (22). According to the report from Debre Birhan, 3.4% of the mothers were concerned about sunning infants, citing pneumonia and blindness were their fears (18). A report from Yirgalem also showed that 5.5% of mothers believed that sunning their children could cause skin cancer and blindness (23). Furthermore, 15.1% of the mothers who participated in a study from Aleta Wondo perceived that sunning could lead to skin cancer, blindness, and sunburn (22). This indicates that mothers were aware of the benefits of infant sunning, yet they were worried about the practice’s safety due to a lack of complete information on how healthy sunning could treat diseases such as pneumonia, skin rash, premature deafness, and blindness (8, 26, 27).

Regarding the duration of sunning, 50.2% of the mothers in the present study exposed their infants for 10–15 min. The finding is higher than the report from Yirgalem (36.5%) (23), Debre Birhan (29.3%) (18), and Debre Markos (38%) (21). In addition, 79.3 and 76.3% of the mothers exposed their infants to sunlight daily and in an unclothed state, respectively. There is also a higher level of practice when compared to previous reports from Yirgalem; 59.9% (23) of mothers exposed their infants to sunlight daily, and only 15.9% of the mothers from Debre Markos (21) exposed their infants to sun unclothe. However, the reports from Debre Birhan, Debre Markos, and Yirgalem were found to be comparable with the present study in terms of the location of sunlight exposure; nearly 90% of the mothers reported outdoor sunning.

On the topic of lubricant application during sunning, 94% of the mothers in the present study used various types of lubricants on the body of their infants, with the majority (88.1%) used butter. Reports from Aleta Wondo (90.8%) (22) and Debre Markos (98.4%) (21), indicated comparable results. However, the findings in Debre Birhan (41.7%) (18) and Yirgalem (63.5%) (23), on the other hand, were found to be lower than the results of the herein study. Likewise, 84.8% of the mothers in this study began sunning their infants before 45 days of neonatal life, which is higher than the reports from Debre Markos (69.5%) (21), Yirgalem (66.4%) (23), and Aleta Wondo (30%) (22). However, it is found to be significantly less than Debre Birhan’s report (99.2%) (18). Further, except for Yirgalem’s report (83%) (23), the current finding is found to be comparable with the findings of Debre Markos (99.1%) (21), Debre Birhan (97%) (18), and Aleta Wondo (90.4%) (23) in regards with the timing of the day to expose infants to be in the morning (8.00–10:00 a.m.).

Concerning the sources of information on the importance of sunning infants, 51.4% of mothers who took part in the present study got the information from health professionals. The finding is lower than the reports from Aleta Wondo (76%) (22) and Debre Birhan (70.5%) (18). However, it is very much higher than the report from Yirgalem (36.5%) (23). Furthermore, 53.5% of mothers from Yirgalem (23) reported that they got the information from neighbors which is incomparable to the findings of the present study and the reports of Debre Birhan and Aleta Wondo (18, 22) as well. However, only 2.4% of mothers accessed the information from the media which is very much lower than the aforementioned reports. The inconsistency could be explained that the setting of this study is rural and the mothers could not access media channels such as TV, radio, and other web-based social platforms.

Among the mothers who participated in the present study, 34.7% of them had a family size of four to six. Nevertheless, 19.8% of them had good knowledge and the odds of knowing the importance of infants’ sunning was 51.1% [AOR = 0.511, 95% CI: (0.352–0.741)] more likely knowledgeable than mothers who had <3 children. The justification could be explained as; birth experiences could improve prior preparedness and awareness of mothers toward infant sunning.

Married women had good practice of infants to sunlight exposure. They were found to be 2.7 times more likely to have good practices than those who got divorced or widowed [AOR = 2.699, 95% CI: (1.131–6.442)]. This finding is found to be incomparable with the report of Debre Markos (21) and Yirgalem (23) in which married women were not significantly associated (*p* > 0.05) with the practice of sunlight exposure. In addition, the odds of having good practice among government-employed mothers were 4 times more likely than those of mothers who were housewives. The finding is also contradictory to the report of Yirgalem; unemployed women were 4.7 times more likely to expose infants to sunlight when compared with the employed (23). Unfortunately, this is in line with the report of Debre Birhan; mothers who were government employees were 5 times [AOR = 5.10, 95% CI: (1.54, 16.92)] more likely to practice good sunlight exposure of their infants than the unemployed (18).

## Conclusion

Although the majority of study participants had a good understanding and practice of infant sunlight exposure, more could be realized through regular maternal health education, women’s involvement in financial as well as social empowerment activities, and encouraging mothers to contact health professionals whenever they have queries of maternal and pediatric-related services. The study’s findings can thus be used as a baseline data source by policymakers and practitioners to develop guidelines and policies aimed at improving pediatric health and related services in and around the study area.

### Strengths and limitations of the study

The large sample size could be taken as the strength of the study. The study comprised roughly one-quarter (25%) of the total estimated target population. Furthermore, the study’s rural location is another aspect that suggests it may be more representative and reflects contemporary reality in a country where more than 85% of the population lives in rural areas. In Ethiopia, the bulk of such studies were undertaken in urban or healthcare settings. The current study’s rural setting and community-based methodology is unique and provided a new data type. However, the study should have included qualitative data; information from focus group discussions (FGD) with selected moms, health professionals, and health leaders to triangulate the quantitative data obtained from interviews.

## Data availability statement

The original contributions presented in the study are included in the article/supplementary material, further inquiries can be directed to the corresponding author.

## Ethics statement

The studies involving human participants were reviewed and approved by Institutional Research Ethics Committee, Mettu University, Mettu, Ethiopia. Written informed consent to participate in this study was provided by the participants. All methods were carried out in accordance with the Helsinki Declaration.

## Author contributions

The study was carried out in collaboration with AT, SY, and AG. AT and SY designed the study, wrote the protocol, and participated in data collection activities. Data analysis was carried out by SY and AG. All authors contributed to the article and approved the submitted version.

## Funding

This paper was conducted with the financial support of the Graduate School of Mettu University, Ethiopia. However, the school has no role in the design of the study, collection, analysis, interpretation of data, and writing the manuscript.

## Conflict of interest

The authors declare that the research was conducted in the absence of any commercial or financial relationships that could be construed as a potential conflict of interest.

## Publisher’s note

All claims expressed in this article are solely those of the authors and do not necessarily represent those of their affiliated organizations, or those of the publisher, the editors and the reviewers. Any product that may be evaluated in this article, or claim that may be made by its manufacturer, is not guaranteed or endorsed by the publisher.

## Footnotes

^1^https://en.wikipedia.org/wiki/Metu_Zuria
